# First confirmed record of *Trichobilharzia franki* Müller & Kimmig, 1994, from *Radix auricularia* (Linnaeus, 1758) for Austria

**DOI:** 10.1007/s00436-020-06938-3

**Published:** 2020-11-05

**Authors:** Susanne Reier, Elisabeth Haring, Florian Billinger, Hubert Blatterer, Michael Duda, Christopher Gorofsky, Hans-Peter Grasser, Wolfgang Heinisch, Christoph Hörweg, Luise Kruckenhauser, Nikolaus U. Szucsich, Alexandra Wanka, Helmut Sattmann

**Affiliations:** 1 Central Research Laboratories, Natural History Museum Vienna, Burgring 7, 1010 Vienna, Austria; 2 Department of Evolutionary Biology, University of Vienna, Althanstraße 14, 1090 Vienna, Austria; 3 Department of Water Management, Office of the State Government of Upper Austria, Kärntnerstraße 12, 4020 Linz, Austria; 4 3rd Zoological Department, Natural History Museum Vienna, Burgring 7, 1010 Vienna, Austria

**Keywords:** First record, *Trichobilharzia*, Schistosomes, Intermediate hosts, Diversity, Swimmer’s itch

## Abstract

Avian schistosomes are of medical and veterinary importance as they are responsible for the annually occurring cercarial dermatitis outbreaks. For Austria, so far, only *Trichobilharzia szidati* Neuhaus 1952 was confirmed on species level as causative agent of cercarial dermatitis. Here we present the first record of *Trichobilharzia franki* Müller & Kimmig 1994 in Austria. The species was detected during a survey of digenean trematodes in Upper Austrian water bodies. Furthermore, we provide DNA barcodes of *T. franki* as well as measurements of several parasite individuals to indicate the intraspecific diversity. We also recommend the usage of an alternative primer pair, since the “standard *COI* primer pair” previously used for Schistosomatidae amplified an aberrant fragment in the sequence of *T. franki*. Overall, our study shows how limited our knowledge about occurrence and distribution of avian schistosomes in Austria is and how important it is to acquire such a knowledge to estimate ecological and epidemiological risks in the future.

## Introduction

Avian schistosomes (Digenea: Schistosomatidae) are globally distributed parasitic flatworms of medical and veterinary relevance. They are characterized by a complex life cycle including freshwater snails as intermediate hosts and birds as final hosts. The cercariae, the free-living infectious stage, may use humans as accidental hosts by penetrating the skin, thus causing cercarial dermatitis (swimmer’s itch), an inflammatory skin disease. An overviewing monitoring of cercarial dermatitis outbreaks in Europe has been provided a few years ago (Soldánová et al. 2013). The causative agents in Europe are mostly species of the genus *Trichobilharzia*, a genus with approximately 35 species worldwide (Brant and Loker 2013), whereof six species are known in Europe, namely *Trichobilharzia szidati* Neuhaus 1952; *Trichobilharzia regenti* Horák, Kolářová, & Dvořák 1998; *Trichobilharzia franki* Müller & Kimmig 1994; *Trichobilharzia salmanticensis* Simon-Vicente & Simon-Martin 1999; *Trichobilharzia anseri* Jouet et al. 2015; and *Trichobilharzia mergi* Kolářová, Skírnisson, Ferté, & Jouet 2013 (Jouet et al. 2010; Horák et al. 2015; Christiansen et al. 2016).

Outbreaks of cercarial dermatitis in Austria have been reported since 1970 (Graefe 1971). In nearly all provinces (except in Vorarlberg), cercarial dermatitis was recorded (Auer and Aspöck 2014). In the first records of schistosomatid cercariae from the lake Neusiedler See in Eastern Austria (Graefe 1971), cercariae shed from *Lymnaea stagnalis* (Linnaeus 1758) were assigned to *T. szidati*. This assignment was based on general morphology and phototactic behavior but lacked confirmation by detailed morphological analyses of either cercariae or adult trematodes. These cercariae were proved to cause dermatitis in humans experimentally and indirectly by records of dermatitis in fishermen and biologists at the site of occurrence of infected snails (Graefe et al. 1973). Cercariae which were gathered in the same study and location from *Planorbarius corneus* (Linnaeus 1758), and assigned to *Bilharziella polonica* (Kowalewski 1895), were also applied to the skin of a test person but did not prove to cause dermatitis (Graefe 1971). Subsequent records of schistosomatid cercariae from different sites in eastern Austria again were assigned to *T. szidati* (Graefe et al. 1973), but with reservation since experimental infections of ducks were not successful. In another study in Eastern Austria, *T. szidati* from *L. stagnalis* was morphologically confirmed by adults from successful infections of ducks, whereas cercariae from *Radix balthica* (Linnaeus 1758) (syn. *Radix ovata* (Draparnaud 1805)) were assigned to the genus *Trichobilharzia* but could not be determined to species level, because snails had died before infection experiments of ducks could be started (Dvořák et al. 1999). More recently, the occurrence of *T. szidati* from *L. stagnalis* from Lower Austria was confirmed by molecular genetic analyses (Gaub 2014).

Additionally, schistosomatid cercariae in Austria had been reported from *Aplexa hypnorum* (Linnaeus 1758), *Gyraulus parvus* (Say 1817), *L. stagnalis*, *Stagnicola* spp., *Radix auricularia* (Linnaeus 1758), and *R. balthica* (syn. *R. ovata*) (Sattmann et al. 2004; Hörweg et al. 2006), yet without species assignment, so the data base for Austria is still incomplete. According to host specificity, it can be assumed that—besides *T. szidati* and *B. polonica*—other avian schistosomes (e.g. *T. franki*, *T. regenti*) may occur in Austria (Auer and Aspöck 2002).

Here, we present the first confirmed record of *T. franki* for Austria detected during a survey of digenean trematodes in Upper Austria. Furthermore, we provided corresponding DNA barcode sequences and compared them with already published haplotypes from different European countries. Besides the new record of *T. franki*, we provide an overview of the status of knowledge concerning avian schistosomes in Austria with implications for future research.

## Material and methods

During a survey of digenean trematodes performed in Upper Austrian water bodies, 229 freshwater snails of different species were collected at Reichersberger Au (European Nature Reserve Lower River Inn; 48.340399 N 13.360308 E; May 27, 2019). Snails were isolated in glasses exposed to daylight and observed for cercarial release. Of those, one individual of *R. auricularia* (out of 10) released schistosomatid cercariae at room temperatures 1 day later. The released cercariae were subsequently put into 80% ethanol for further analyses. Five specimens were measured and documented and deposited in the collection Evertebrata varia of the NHMW, and another five specimens were analyzed genetically. Since the latter specimens were completely consumed for the genetic analysis, the five preserved specimens serve as para-vouchers.

### Morphological analysis

For determination, we measured body length and width, stem length and width, and furca length of five specimens of the released cercariae in *NIS elements* (Nikon Instruments Inc., New York, USA). Microphotographs were taken with a Nikon Eclipse Ni-U microscope equipped with a Nikon DS-Ri2 microscope camera.

### Molecular genetic analysis

DNA extraction was performed in a clean room with the *QIAmp DNeasy Blood and Tissue Kit* (QIAGEN, Hilden, Germany) by following the protocol of the manufacturer. To perform the final elution step in 15 μl AE buffer we used *QIAmp MinElute* columns of the *QIAamp DNA Micro Kit* (QIAGEN, Hilden, Germany).

A partial sequence of the mitochondrial cytochrome *c* oxidase subunit 1 (*COI*) gene was amplified using the primers SchistoCox1-5′ (5′-TCT TTR GAT CAT AAG CG-3′) and SchistoCox1-3′ (5′-TAA TGC ATM GGA AAA AAA CA-3′) amplifying a 1125-bp PCR fragment (Lockyer et al. 2003). PCR reactions were performed in a final volume of 25 μl containing 15.9 μl distilled water, 2.5 μl 10× PCR buffer, 1.5 mM MgCl_2_, 0.2 mM of each dNTP, 0.5 μM of each primer, 0.5 units *TopTaq* DNA Polymerase (QIAGEN, Hilden, Germany), and 3 μl template DNA. PCR amplification was performed under the following conditions: 95 °C for 2 min, 40 cycles of (95 °C for 60 s, 52 °C for 60 s, and 72 °C for 120 s) and 72 °C for 7 min. We designed an alternative internal primer ZDOE-COI-rv (5′-TAGTTTRTTTCATGATACTTG-3′), which was used in combination with SchistoCox1-5′ and amplified a 1058-bp fragment under the same PCR conditions as described above. The PCR products were sequenced (both directions) by Microsynth (Balgach, Switzerland) using the PCR primers.

The five *COI* sequences determined in the present study did not show any sign of nuclear pseudogenes (e.g., insertions/deletions or nonsense mutations) and were deposited in Barcode of Life Data Systems (BOLD) and GenBank under the accession numbers CDOE-001-20–005-20 (BOLD) and MT763194–MT763198 (GenBank).

All available *COI* sequences of the genus *Trichobilharzia* in GenBank and BOLD were batch downloaded using the package PrimerMiner (Elbrecht and Leese 2017) in R 3.6.3 (R Core Team 2018). Subsequently, sequences were loaded into Geneious 2.10.3 (https://www.geneious.com) and aligned using MAFFT (Katoh and Standley 2013). The alignment was trimmed to 665 bp, and all sequences shorter than this threshold were excluded from the final alignment. The final alignment contained 120 sequences, including the five sequences processed in this study. ModelFinder (Kalyaanamoorthy et al. 2017) implemented in PhyloSuite (Zhang et al. 2020) was used to select the best-fit model (GTR+F+I+G4) using BIC criterion. Bayesian inference was conducted using MrBayes 3.2 (Ronquist et al. 2012) with two runs, each having four chains, and run for 5 × 10^6^ generations each. Trees and parameters were sampled every 250th generation. After discarding the first 25% of trees as burn-in, a 50% majority rule consensus tree was built from the remaining trees.

A median-joining haplotype network (Bandelt et al. 1999) using PopART 1.7 (http://www.popart.otago.ac.nz) was produced for the species *T. franki* to illustrate the variability of specimens in Austria among other specimens from Europe. Therefore, *COI* sequences of *T. franki* from different European countries (accession numbers HM131197–HM131205, FJ174530) were included into the alignment. Due to different lengths of the sequences, we trimmed the final alignment to a length of 682 bp. Networks were graphically processed in InkScape 0.92 (https://inkscape.org). The haplotypes were classified according to their collection countries. Haplotype diversity (Hd) and nucleotide diversity (π) were calculated in DnaSP v5 (http://www.ub.edu/dnasp; Librado and Rozas 2009).

## Results

### Morphology

The following measurements (given as means with standard deviation in brackets) were obtained from five specimens of *Trichobilharzia* cercariae released from one *R. auricularia* individual: body length 320 μm (± 14.2), body width 62 μm (± 1.6), tail stem length 409 μm (± 6.2), tail stem width 47 μm (± 3.0), and furca length 233 μm (± 4.2).

A comparison of our results with already published measurements of *Trichobilharzia* spp. (Müller and Kimmig 1994; Podhorský et al. 2009; Jouet et al. 2010) revealed variations within and between species and highlight the difficulties and limitations of a solely morphological determination (Table 1). Although measured body lengths of our specimens fall in the range of measures for *T. franki* from the mentioned previous studies, there is an overlap in size between all species (Table 1). Nevertheless, most of our measurements fall in the range of the measurements of the original species description of *T. franki* (Table 1; Müller and Kimmig 1994).

**Table 1:** Measurements (in μm, means) of *T. franki* of this study compared with measurements of *T. franki*, *T. szidati*, *T. regenti*, and an undetermined species from previous studies (Müller and Kimmig 1994; Podhorský et al. 2009; Jouet et al. 2010). Standard deviations are given if available. Abbreviations: BL, body length; BW, body width; TSL, tail stem length; TSW, tail stem width; FL, furca length

Species	Number	Host	BL	BW	TSL	TSW	FL
*T. franki* ^1^	5	*R. auricularia*	320 ± 14.2	62 ± 1.6	409 ± 6.2	47 ± 3.0	233 ± 4.2
*T. franki* ^2^	26	*R. auricularia*	307 ± 10.4	71.5 ± 2.7	419 ± 11.9	48.4 ± 3.0	234 ± 16.5
*T. franki* ^3^	55	*R. auricularia*	318	81	427	56	273
*T.* sp. ^3^	31	*R. labiata*	257	73	379	50	227
*T. franki* ^4^	64	*R. auricularia*	316 ± 14	67 ± 7	480 ± 30	55 ± 5	318 ± 18
*T. szidati* ^4^	251	*L. stagnalis*	293 ± 36	63 ± 12	399 ± 40	47 ± 6	273 ± 30
*T. regenti* ^4^	124	*R. labiata*	333 ± 48	78 ± 14	421 ± 31	59 ± 9	285 ± 23

^1^This study

^2^Müller and Kimmig (1994)

^3^Jouet et al. (2010)

^4^Podhorský et al. (2009)

### Molecular genetic analyses

When sequencing the 3′ end of the amplicon generated of the five cercariae analyzed using the primer pair previously used in other studies (SchistoCox1-5′/SchistoCox1-3′), we faced difficulties: The reverse read (sequencing primer SchistoCox1-3′) delivered a mixed sequence downstream of site 1000 in the alignment; there were many, albeit small, double peaks, which occurred also after the repetitions of sequencing. Although, no similarity was found in this section with the expected reference sequences, a BLAST search of this aberrant fragment showed that it was clearly similar to *T. franki* (97% identity score). Further examination revealed that this result was due to an additional internal primer binding of the primer SchistoCox1-3′ in the 5′ part of the *COI* gene (sites 123 to 142 of the alignment). Consequently, in the sequencing reaction, two amplicons of different lengths were sequenced simultaneously. We did overcome this problem by designing a new reverse primer (ZDOE-COI-rv) to exclude this unintended unspecific binding. This primer was used as PCR primer and as sequencing primer. The resulting amplicon (amplified in combination with primer SchistoCox1-5′) is 67 bp shorter (1058 bp).

**Fig. 1:**
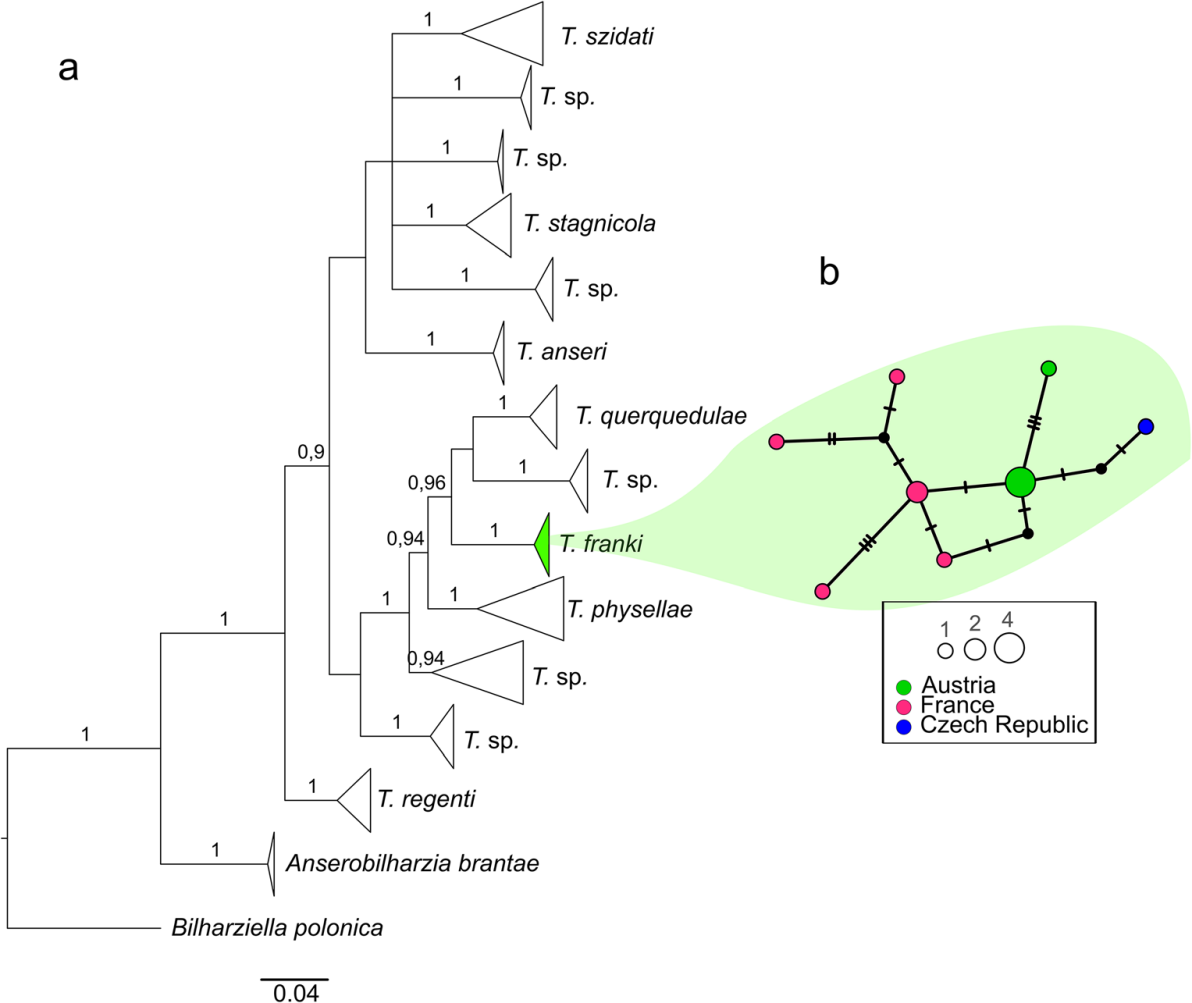
Phylogenetic relationships between species of *Trichobilharzia*. **a** Bayesian inference (BI) tree including 120 *COI* sequences of different species of *Trichobilharzia*. Only Bayesian probabilities ≥ 0.9 are given next to the nodes. The clade containing sequences of *Trichobilharzia franki* processed in this study is colored. **b** Median-joining network (MJ) of *COI* sequences of *T. franki* from different European countries. Haplotypes are constituted of one to four samples (see legend). Sequences within the haplogroups are separated by one to three mutation steps. Mutation steps are indicated by vertical lines. Black dots represent missing haplotypes

The BI tree revealed two main clusters of *Trichobilharzia* spp. The first cluster contains *T. szidati*, *T. stagnicolae*, and *T. anseri* and three clades of undetermined sequences of *Trichobilharzia*. The second main clade includes sequences of *T. franki*, *Trichobilharzia querquedulae* McLeod 1937, *Trichobilharzia physellae* (Talbot 1936) McMullen & Beaver 1945, and three clades of undetermined sequences of *Trichobilharzia*. The five specimens processed in this study cluster together with sequences from GenBank determined as *T. franki* (Fig. 1a). Most importantly, the sequences of this study are clearly distinguished from *T. szidati*, until now the only species of *Trichobilharzia* known from Austria. The second species expected in Austria, *T. regenti*, falls outside the two main clades of *Trichobilharzia* spp. as well as *Anserobilharzia brantae* (Brant et al. 2013) (Fig. 1a). To summarize, the results of the *COI* sequence comparisons clearly confirmed the tentative morphological assignment to *T. franki*

The network in Fig. 1 shows genetic diversity of the *COI* sequences determined in the present study in more detail. Among the five Austrian specimens of *T. franki*, two *COI* haplotypes were present, separated by three mutation steps (Fig. 1b). The additional seven *COI* sequences of *T. franki* from other European countries contained six haplotypes (Fig. 1b). We identified a considerable high haplotype diversity (Hd 0.97) and a nucleotide diversity (*π*) of 0.04. The similarities among and within the sequences of the different countries are low, and no clearly separated geographic haplogroups were distinguished. Furthermore, no haplotype sharing among individuals from different countries was observed.

## Discussion

As outlined above, the knowledge of *Trichobilharzia* spp. occurring in Austria is low (Dvořák et al. 1999; Sattmann et al. 2004; Hörweg et al. 2006) despite of their medical and veterinary importance. Thus, it is essential to determine the diversity of avian schistosomes, as well as their distribution, occurrence, and intermediate and final host range (Brant and Loker 2013).

In this study, we present the first confirmed record of *T. franki* in Austria. Species assignment was straightforward combining DNA sequence comparisons with morphological features. It has long been suspected that, apart from *T. szidati*, also other representatives of the genus occur in Austria (Auer and Aspöck 2002). *Trichobilharzia franki* was first described from Southern Germany (Müller and Kimmig 1994) and subsequently has been reported from many European countries (reviewed in Jouet et al. 2010) but was not yet detected in Austria. The first record of *T. franki* in Austria shows the importance of DNA-based methods in combination with classical morphological analyses. Morphological diversity of *T. franki* is reflected in the variation of body measurements evaluated in different studies (Müller and Kimmig 1994; Podhorský et al. 2009; Jouet et al. 2010) and was found also in the present study (Table 1). The variation in measurements might to some extent be caused by the contractibility of the body of cercariae of avian schistosomes (Podhorský et al. 2009; Jouet et al. 2010). Furthermore, measurements might also vary because of different fixation and preservation conditions. These factors may, in addition to the true morphological diversity, hamper a reliable identification (Jouet et al. 2010), wherefore we do not suggest species assignment based solely on morphological characters. Also, environmental and/or host-related factors may influence the size of cercariae, e.g. temperature (Dönges 1964) or snail age (Neuhaus 1952).

Podhorský et al. (2009) conducted morphological comparisons between cercariae of *T. szidati*, *T. franki*, and *T. regenti* and concluded that the species could be differentiated only by specific distribution of sensory papillae, but not by cercarial body dimensions. In the present study, due to our workflow of sampling and studying, we were not able to study the papillae. Therefore, to enable a reliable taxonomic assignment, DNA sequence analysis and comparison with published sequences is crucial. Sequences that were derived from determined laboratory strains are important as they allow a reliable assignment based on DNA sequences.

To date, there are only very few published *COI* sequences (*n* = 10) of *T. franki* available, while mostly sequences of the ribosomal RNA gene cluster (mostly internal transcribed spacer 1 and 2 (*ITS1* and *ITS2*)) were analyzed. Nevertheless, studies, where *COI* and *ITS* sequences were generated from the same specimens (Jouet et al. 2010), enable a comparison with a large database of *T. franki* sequences in GenBank. Thus, we can trust the assignment of *COI* sequences, although the number of comparison sequences is comparatively low.

We found a high haplotype diversity of 0.97 among the analyzed sequences of *T. franki*, similar to the diversity detected in previous studies for the species *T. franki*, *T. szidati*, and *T. regenti* (Lopatkin et al. 2010; Korsunenko et al. 2012). The population structure of parasites is often shaped by the migration of the final hosts (Jarne and Theron 2001), which are anatid birds in the case of *Trichobilharzia* spp. The avian mobility might enable gene flow between parasites populations even over large distances (Korsunenko et al. 2012). The presence of two haplotypes in cercariae from one snail is an interesting finding, which suggests that snail individuals may be infected by several miracidae from different origins, which then co-exist and propagate within one individual.

The establishment of a DNA barcode reference database will be of great advantage in the identification of these avian schistosomes. With one exception (Gaub 2014), molecular genetic methods had so far never been applied in previous studies on Austrian schistosomatid cercariae. On an international scale, available data are also scarce. The problems we faced with unspecific primer binding exemplify the problems that widely used “universal” primers may cause. Sequence comparison showed that the unspecific binding had eight mismatches (out of 20 sites) in *T. franki* and still worked well as a sequencing primer (in addition to the correct binding site). Once both primer binding sites were detected, both sequences could be readily distinguished as the sequence derived from the internal site produced largely higher peaks resulting in a well readable sequence. The more reliable DNA barcodes are available, the better are the prerequisites for further studies: for straightforward taxonomic assignment, for detection of presumably cryptic species, for additional primer design, as well as for critical data evaluation.

At the present state of knowledge, it is not recommended to determine species of *Trichobilharzia* based on presumed host specificity, especially since reports in the literature regarding host range are not consistent. The specimens identified in the present study parasitized *R. auricularia*, the type host of *T. franki.* Yet, in some studies, also *R. labiata* (Rossmässler, 1835) (syn. *Radix peregra* [O. F. Müller 1774]) (Aldhoun et al. 2009; Jouet et al. 2010) and even *L. stagnalis* (Rudolfovà et al. 2005) have been reported as intermediate hosts of *T. franki*. Nevertheless, these results must be considered with caution. High genetic differences were detected by Jouet et al. (2010) between specimens of *T. franki* obtained in France from *R. labiata* and from *R. auricularia*, which suggested that the specimens obtained from *R. labiata* were a hitherto undetected cryptic species. Furthermore, the considerably high number of undetermined clades containing species of *Trichobilharzia* found in the BI tree (Fig. 1a) suggest that more cryptic species exist. In general, it has been shown that more cryptic species tend to be uncovered among trematodes compared to other helminth taxa (Pérez-Ponce de León and Poulin 2018), which might not only be due to the frequent lack of suitable morphological structures or their complex life cycles but also to the way in which trematode species are described. In a previous study in Austria, an unidentified species of *Trichobilharzia* was obtained from *R. balthica* (syn. *R. ovata*) (Dvořák et al. 1999). Two scenarios are plausible regarding the species assignment of this unidentified species: (1) it was *T. franki*, which could not be identified properly by morphology. (2) It is another previously unknown species or a species so far not detected in Austria. Therefore, to aim at a complete inventory of *Trichobilharzia* spp. in Austria, intensive sampling of a broader geographic range covering the known distribution and considering more potential intermediate hosts is required. There is a high probability that also *T. regenti* occurs in Austria, which also uses *Radix* spp. as intermediate hosts.

## Conclusion

Besides the first report of *T. franki* in Austria, we provided further insights of intraspecific morphological and genetic diversity. Moreover, the study showed that the analysis of digenean trematodes with complex life cycles is extremely challenging, both morphologically and genetically. Therefore, an integrative taxonomic approach is essential to identify species and to assess their distribution, since more species can be expected to occur in Austria. As soon as a database including determined specimens is established, the usage of environmental DNA (eDNA) for the monitoring of cercarial dermatitis outbreaks can be applied in addition to conventional methods. Eventually, putting all the information from various countries together will allow to accurately assess distribution ranges and species diversity. The more we know, the better we are prepared regarding epidemiological and ecological risks, efficient control of dermatitis outbreaks, and potential future changes in parasite composition.
